# Local Experience with Extracorporeal Membrane Oxygentaion in Children with Acute Fulminant Myocarditis

**DOI:** 10.1371/journal.pone.0082258

**Published:** 2013-12-09

**Authors:** Botao Ning, Chenmei Zhang, Ru Lin, Linhua Tan, Zhenjie Chen, Jia Yu, Tao Liu, Zihao Yang, Sheng Ye

**Affiliations:** Pediatric Intensive Care Unit, Children’s Hospital, Zhejiang University School of Medicine, Hangzhou, Zhejiang Province, China; Merck & Co., United States of America

## Abstract

To analyze the clinical effect of extracorporeal membrane oxygenation (ECMO) in children with acute fulminant myocarditis, we retrospectively analyzed the data of five children with acute fulminant myocarditis in the intensive care unit (ICU) at the Affiliated Children’s Hospital, Zhejiang University from February 2009 to November 2012. The study group included two boys and three girls ranging in age from 9 to 13 years (median 10 years). Body weight ranged from 25 to 33 kg (mean 29.6 kg). They underwent extracorporeal membrane oxygenation (ECMO) through a venous-arterial ECMO model with an average ECMO supporting time of 89.8 h (40–142 h). Extracorporeal circulation was established in all five children. After treatment with ECMO, the heart rate, blood pressure, and oxygen saturation were greatly improved in the four children who survived. These four children were successfully weaned from ECMO and discharged from hospital machine-free, for a survival rate of 80% (4/5). One child died still dependent on the machine. Cause of death was irrecoverable cardiac function and multiple organ failure. Complications during ECMO included three cases of suture bleeding, one case of acute hemolytic renal failure and suture bleeding, and one case of hyperglycemia. During the follow-up period of 4–50 months, the four surviving children recovered with normal cardiac function and no abnormal functions of other organs. The application of ECMO in acute fulminant myocarditis, even in local centers that experience low incidence of this disease, remains an effective approach. Larger studies to determine optimal timing of placement on ECMO to guide local centers are warranted.

## Introduction

Acute fulminant myocarditis is a serious disease with rapid progression. It can be reversed with recovery of normal ventricular function if effective adjunctive treatment is applied early, addressing the damaged cardiopulmonary and end organs. The patient can recover myocardial function. When medications fail to reverse the patient’s clinical course, extracorporeal membrane oxygenation (ECMO) is considered the most effective supportive and adjunct strategy [1,2].

The principle of ECMO is that it supports, and acts as an adjunct to, the failing respiratory and circulatory systems. The mechanism of ECMO is that the blood is driven through the membrane oxygenator by a pump for oxygenation and then transported back to the patient. ECMO provides simultaneous support for both right and left ventricles and can replace pulmonary function. It thereby provides stable circulatory blood volume while effectively maintaining blood and oxygen supplies for important organs such as the heart and brain in patients with cardiopulmonary failure, allowing the patient’s heart and lungs to rest [3].

In 1972, Hill successfully performed venous-arterial (V-A) ECMO therapy for the first time [4]. Thereafter, ECMO was promptly applied in the clinical realm, producing good effects. This article summarizes the clinical application of ECMO in five children with acute fulminant myocarditis and discusses its effects.

## Materials and Methods

### Patients

The patients in the study included five children with acute fulminant myocarditis admitted to the pediatric intensive care unit (PICU) of the Affiliated Children’s Hospital, Zhejiang University School of Medicine from February 2009 to November 2011. All five children were treated with adjunctive ECMO therapy. This study group included two boys and three girls ranging in age from 9 to 13 years (median 10 years). Their mean body weight was 29.6 kg (range 25–33 kg). The ECMO treatment period was 40–142 h (mean 89.8 h). Among the five children, two had serious circulatory failure and three had cardiac arrest. Three of the children underwent ECMO with simultaneous cardiopulmonary resuscitation (CPR).

### Methods

 All the content was approved by the patients’ parents and the Ethics Committee of Children’s Hospital of Zhejiang University School of Medicine. We did obtain informed and written consent from the next of parents on the behalf of the children participants involved in our study, and the consents were documented in the hospital computer system. The above consent procedure was approved by the ethics committees.

The patients were diagnosed as acute fulminant myocarditis according to the criteria: (1) severe and acute heart failure or/and cardiogenic shock; (2) left ventricular dysfunction assessed by echocardiography; (3) abnormal ECG, such as ST elevation, T inversion, and conduction block; (4) increasement of creatinine kinase (CPK-MB) and cardiac troponin; (5) a recent history of viral infection; and (6) absence of personal or familial history of cardiomyopathy. Serious circulatory failure was defined as severe systemic hypotension requiring maintainance of inotropic medicines or due to life threatening arrhythmias.

The ECMO was established in the PICU with kits (Maquet, Rastatt, Germany) that included a centrifugal pump, membrane oxygenator, air and oxygen mixing device, oxygen saturation probe, and circulation tubes. All five patients were administered V-A ECMO. Heparin (1 mg/kg) was given 3 min prior to intubation. After establishing the ECMO, it was adjusted based on the activated clotting time (ACT) to maintain the ACT at 140–220 seconds. During the maintenance period on ECMO, the dosages of vasoactive medicines were reduced gradually to null. Respiratory support was administered as synchronized intermittent mandatory ventilation. The FiO_2_ was 30–45%, breathing frequency was 10–15/min, tidal volume was half of the normal amount, and the positive end-expiratory pressure (PEEP) was 4–5 cmH_2_O. The adjunct flow volume was adjusted according to the hemodynamic indices and was maintained at 50–150 ml/min/kg to maintain venous oxygen saturation at >65%. The FiO_2_ of the air and oxygen mixture entering the membrane lung was kept at 40–60% and arterial oxygen saturation at ≥95%. The circulation and respiration conditions during ECMO were evaluated using daily echocardiography, chest radiography, blood gas analyses, and measurements of the patient’s hemodynamic state. When the cardiac function recovered to baseline with enough storage, the adjunct flow volume was gradually reduced, vasoactive medicines were increased, and the ACT was properly prolonged. When the adjunct flow volume had been reduced to 10–20% of the total flow volume, ECMO was removed for an observation trial of 30 min. If the patient’s respiration was stable, the cannula was removed. The cervical vessel was ligated, and the femoral artery and vein were repaired.

## Results

Large dosages of vasoactive medicines including epinephrine (0.2–1.0 μg/kg∙min), dopamine (10–15 μg/kg∙min), and/or dobutamine (10–20 μg/kg∙min) were administered before ECMO, but they failed to improve the blood pressure, then ECMO was established immediately on the five patients, at the same time, respirator was performed on each patient. After ECMO establishment, the heart rate, blood pressure, and oxygen saturation were obviously improved (Table 1).

**Table 1:** Comparison of indices before and after ECMO.

Indices	Case 1		Case 2		Case 3		Case 4		Case 5	
	Before	After	Before	After	Before	After	Before	After	Before	After
Heart rate (bpm)	160	100	40	85	15	72	52	80	170	90
Systolic pressure (mmHg)	65	110	60	95	45	85	50	90	55	100
CVP (cmH_2_O)	10	8	12	10	13	9	12	10	14	8
SPO_2_ (%)	65	>95	55	>95	35	>95	65	>95	70	>95

CVP: central venous pressure; ECMO: extracorporeal membrane oxygenation; SPO_2_: transcutaneous oxygen saturation

Venous-arterial ECMO was established in all five children, with an average support duration of 89.8 h (40–142 h). Four of the five children were weaned successfully from the ECMO and were discharged from the hospital machine-free. One child died from withdrawal of ECMO support, who was unable to wean from ECMO and the respirator due to irrecoverable cardiac function and complicated multiple organ failure. The survival rate was therefore 80% (4/5).

The major complications during ECMO maintenance were bleeding at the sutures in three cases, hemolytic renal failure and suture bleeding in one case each, and hyperglycemia in one case. The overall follow-up was 4–50 months. The cardiac function of the 4 survivors, pre-ECMO, at the time of hospital discharge and at the follow-up visits was listed (Table 2). All four of the surviving children recovered normal cardiac function, with no abnormal function in other organs (Table 3).

**Table 2:** The cardiac function of the 4 survivors pre-ECMO, at the time of hospital discharge and at the follow-up visits.

Patients	Pre-ECMO		Hospital discharge		1 month after discharge		3 months afer discharge	
	LVEF	LVFS	LVEF	LVFS	LVEF	LVFS	LVEF	LVFS
1	25	18	57	31	65	34	68	37
2	23	16	60	33	69	36	61	33
4	22	15	58	31	67	37	65	32
5	29	19	65	34	62	32	71	38

LVEF: lefr ventricular ejection fraction; LVFS: lefe ventricular fractional shortening.

**Table 3:** Clinical data for five children with acute fulminant myocarditis treated with ECMO.

#	Sex	Age (years)	Weight (kg)	Diagnosis	Model	Duration (hs)	Complications	Outcomes	Sequel
1	Male	10	32	AFM, serious circulation failure	Left femoral vein–right femoral artery	140	Suture bleeding	Survival	No
2	Male	10	30	AFM, cardiac arrest	Right femoral vein–right femoral artery, E-CPR	40	Suture bleeding	Survival	No
3	Female	9	25	AFM, cardiac arrest	Right femoral vein–right femoral artery, E-CPR	55	Suture bleeding Hemolytic renal failure	Death	
4	Female	9	28	AFM, cardiac arrest	Right jugular vein–right cephalic artery, E-CPR	142	Hyperglycemia	Survival	No
5	Female	13	33	AFM, serious circulation failure	Right femoral vein–right cephalic artery	72	Suture bleeding	Survival	No

AFM: acute fulminant myocarditis

## Discussion

Although there have been numerous reports about acute fulminant myocarditis, it is still difficult to classify myocarditis [5]. The America Heart Association recommended an endocardial biopsy for patients with myocarditis, but the test is not yet performed routinely in those with suspected myocarditis [6]. The existence of inflammatory cells in the heart and cardiac necrosis has prognostic significance but is not necessarily a requirement for diagnosing myocarditis. Although autopsy examinations can detect patchy myocardial damage, biopsy of the endocardium is not always helpful. Some myocardiotropic viruses (e.g., Coxsackie virus) easily cause patchy myocardial damage, whereas others (e.g., adenovirus) create myocardial damage that is visible only with light microscopy—even though all virus-induced damage can take on a fulminant course [7]. Although many polymerase chain reaction examinations of serum and tissues and serology studies have been performed, no specific pathogen was found in most patients [8]. Therefore, the myocardial damage caused by fulminant myocarditis may result from multiple factors that determine the progression of the disease. Some researchers have even questioned whether infection is a necessary condition for myocardial damage or if the myocardial damage is the result of an autoimmune reaction [9].

The mortality rate for acute fulminant myocarditis is high—up to 50% without ECMO support [10]. In the present study, all five children with acute fulminant myocarditis were provided with timely ECMO support, and four children survived, resulting in the high survival rate of 80%, which is much higher than that with regular immune therapy.

Although not all patients with arrhythmia or end-organ failure require ECMO support, their circulation function may stop functioning at any time. Hence, clinical understanding of the condition is required to make the correct decision about treatment. Most of the literature lists arrhythmia, end-organ failure, and circulation failure as indications of ECMO support [11,12]. For patients with acute fulminant myocarditis requiring ECMO, intubation and therapy decisions must be prompt [13]. We sent the five children described here directly to the PICU and performed bedside intubation. Regarding the three children who underwent CPR in the present study, there is only a small possibility of stabilizing the circulation after CPR. In addition, long-term CPR can significantly increase the risk of nervous system damage. Considering the malignant level of fulminant myocarditis and the low risk of mechanical assistance at an early stage, the choice of ECMO is reasonable for patients with arrhythmia and end-organ and circulation failure.

There are two types of ECMO [14]: (1) venous-venous (V-V) ECMO, in which drained venous blood is oxygenated in an oxygenator, the carbon dioxide is removed, and the oxygenated blood is pumped back into the body via another vein. The rationale is to partially exchange gases in venous blood before the blood flows through the lungs to make up for insufficient pulmonary function. V-V ECMO is suitable for patients with simple pulmonary function damage but without risk of cardiac arrest (2). V-A ECMO, in which drained venous blood is oxygenated in an oxygenator, carbon dioxide is removed, and the oxygenated blood is pumped into an artery. V-A ECMO simultaneously assists cardiac and pulmonary functions. It is suitable for patients with cardiac failure, serious pulmonary failure, and possibly cardiac arrest. The five children with acute fulminant myocarditis in the present study underwent V-A ECMO. During ECMO support, we found that the incidence rate of bleeding was the most, which was in accordance with the report of Almond [15].

The survival rate for children with acute fulminant myocarditis who undergo ECMO is closely related to the severity of the disease [16]. In the present study, the patient who died was in poor general condition before ECMO assistance, although the child experienced hemolytic renal failure during ECMO. The heart rate was 15 beats/min, the systolic blood pressure 45 mmHg, and the SPO_2_ 35%. In all, 80% of our acute fulminant myocarditis patients who underwent ECMO recovered normal cardiac function. This total survival rate was higher than those reported by Teele and Almond [15,17].. The major reason may be the small sample of only five cases. In addition, data from the Extracorporeal Life Support Organization indicated that long-term ECMO (>12.5 days) increased the mortality rate for patients with cardiac disease [18]. Amond also concluded that duration of ECMO > 14 days was one of the patient factors predicting nonsurvial, and ECMO may be effective for short-term circulatory support [15]. In the present study, the mean ECMO duration was <4 days (89.8 h), suggesting that short-term ECMO support may be the bridge for increasing the survival rate of patients with fulminant myocarditis.

Cardarelli et al. reported that mortality associated with ECMO for adults in cardiac arrest (E-CPR) reached 40% [19]. In the present study, three patients underwent E-CPR and two survived, for a survival rate of 66.7%, which was higher than has previously been reported. The discrepancy might be related to the small sample, the time window for establishing ECMO, and the efficiency of the CPR.

ECMO can effectively support children with reversible cardiopulmonary failure as it improves oxygenation, removes carbon dioxide, maintains stable hemodynamics, and supports recovery of cardiopulmonary functions. The application of ECMO in acute fulminant myocarditis, even in local centers that experience low incidence of this disease, remains an effective approach. Larger studies to determine optimal timing of placement on ECMO to guide local centers are warranted.
